# Causes of death in individuals with lifetime major depression: a comprehensive machine learning analysis from a community-based autopsy center

**DOI:** 10.1186/s12888-024-05946-2

**Published:** 2024-07-24

**Authors:** Paula Villela Nunes, Livia Mancine, Beatriz Astolfi Neves, Renata Elaine Paraizo Leite, Camila Nascimento, Carlos Augusto Pasqualucci, Beny Lafer, Rogerio Salvini, Claudia Kimie Suemoto

**Affiliations:** 1 Faculdade de Medicina de Jundiai, rua Francisco Telles, 250, Jundiai, SP 13202-550 Brazil; 2 Faculdade de Medicina, Universidade de Sao Paulo, Av. Dr. Arnaldo, 455, Sao Paulo, SP 01246-903 Brazil; 3 Instituto de Informatica Universidade Federal de Goias, Alameda Palmeiras, s/n, Goiania, GO 74690-900 Brazil; 4 Instituto Federal Goiano, Campus Ceres, GO-154, km 218, zip, Ceres, GO 76300-000 Brazil; 5 Federal University of Sao Paulo, rua Pedro de Toledo, 669, Sao Paulo, SP 04039-032 Brazil

**Keywords:** Mortality, Autopsy, Depression, Machine learning, Death cause

## Abstract

**Background:**

Depression can be associated with increased mortality and morbidity, but no studies have investigated the specific causes of death based on autopsy reports. Autopsy studies can yield valuable and detailed information on pathological ailments or underreported conditions. This study aimed to compare autopsy-confirmed causes of death (CoD) between individuals diagnosed with major depressive disorder (MDD) and matched controls. We also analyzed subgroups within our MDD sample, including late-life depression and recurrent depression. We further investigated whether machine learning (ML) algorithms could distinguish MDD and each subgroup from controls based on their CoD.

**Methods:**

We conducted a comprehensive analysis of CoD in individuals who died from nontraumatic causes. The diagnosis of lifetime MDD was ascertained based on the DSM-5 criteria using information from a structured interview with a knowledgeable informant. Eleven established ML algorithms were used to differentiate MDD individuals from controls by simultaneously analyzing different disease category groups to account for multiple tests. The McNemar test was further used to compare paired nominal data.

**Results:**

The initial dataset included records of 1,102 individuals, among whom 232 (21.1%) had a lifetime diagnosis of MDD. Each MDD individual was strictly paired with a control non-psychiatric counterpart. In the MDD group, the most common CoD were circulatory (67.2%), respiratory (13.4%), digestive (6.0%), and cancer (5.6%). Despite employing a range of ML models, we could not find distinctive CoD patterns that could reliably distinguish individuals with MDD from individuals in the control group (average accuracy: 50.6%; accuracy range: 39-59%). These findings were consistent even when considering factors within the MDD group, such as late-life or recurrent MDD. When comparing groups with paired nominal tests, no differences were found for circulatory (*p*=0.450), respiratory (*p*=0.790), digestive (*p*=1.000), or cancer (*p*=0.855) CoD.

**Conclusions:**

Our analysis revealed that autopsy-confirmed CoD exhibited remarkable similarity between individuals with depression and their matched controls, underscoring the existing heterogeneity in the literature. Future research should prioritize more severe manifestations of depression and larger sample sizes, particularly in the context of CoD related to cancer.

**Supplementary Information:**

The online version contains supplementary material available at 10.1186/s12888-024-05946-2.

## Introduction

Depression is a prevalent and disabling mental disorder that frequently co-occurs with a wide range of chronic conditions. It is associated with elevated all-cause mortality and is an established risk factor for completed suicide. The evidence of the association between depression and suicide is unquestionable. Nevertheless, within the demographic of individuals grappling with depression, suicide seems to represent a comparatively smaller proportion of fatalities when contrasted with deaths resulting from natural causes [1]. Although depression is associated with an increased risk of all-cause mortality, there is still divergence in the literature on specific causes of death [1–4].

Multiple factors and mechanisms may contribute to the associations between depression and all-cause mortality. As an example of a direct effect, depression activates several mechanisms that could contribute to the emergence of chronic somatic diseases that are consistently related to decreased survival. For instance, depression is associated with increased peripheral inflammation and oxidative stress, which may contribute to the associations between depression and obesity and cardiometabolic conditions, including obesity and this effect is usually more pronounced in recurrent depression (RD) [1, 3]. Late-life depression (LLD) is often considered a distinct depression type, with more cardiovascular and atherosclerotic associated burden [5] and increased risk for dementia [6]. Moreover, a few conditions may have an unexpected shared genetic basis with depression [7]. As an example of the indirect effects of depression, depression may alter illness behavior, leading to decreased adherence to treatment and unhealthy lifestyles [4]. Finally, depression often coexists with other mental health conditions that may also be associated with increased mortality rates, such as alcoholism [1, 3].

The associations of depression with all-cause and specific causes of mortality have been investigated in different settings. The data are usually derived from prospective studies or health databases and from death reports. Major depressive disorder (MDD), for instance, may confer a higher risk for several noncommunicable diseases associated with increased mortality (e.g., diabetes, obesity, stroke, acute myocardial infarction, dementia, and physical health multimorbidity); however, these chronic health conditions also appear to increase the likelihood of developing depression [1]. However, the evidence becomes weaker when focusing on studies that used structured interviews and those adjusted for potential confounders, including comorbid conditions. As a result, the evidence for causal associations between depression and all-cause mortality remains inconclusive [1, 2].

Autopsy studies have proven to be a valuable source of detailed information on pathological conditions or underreported ailments [8]. Surprisingly, there is a lack of research examining the diverse causes of death (CoD) among individuals with depression using comprehensive full-body autopsy reports.

Machine learning (ML) is a method of data analysis that automates analytical model building and provides a distinct and often complementary analysis to methods commonly used in health sciences. It is a branch of artificial intelligence based on the idea that systems can learn from data, identify patterns, and make decisions with minimal human intervention. ML is particularly appropriate to analyze complex datasets and to model non-linear relationships between variables, allowing for more nuanced predictions [9].

Therefore, this study aimed to investigate whether MDD was associated with specific CoD based on full-body autopsy reports in a large multiethnic community-based sample. We also analyzed subgroups within our MDD sample, which included individuals with LLD and more severe cases, such as RD. We further investigated whether machine learning (ML) algorithms could distinguish MDD and each subgroup from controls based on their CoD.

## Materials and methods

### Participants

Participants were deceased individuals who underwent autopsy at the Sao Paulo Autopsy Service (SVOC) and whose brains were donated to the Biobank for Aging Studies of the University of Sao Paulo (BAS-USP) [5, 10, 11] from 2004 to 2019. SVOC is a community-based autopsy service for individuals who die from natural (nontraumatic) causes. The SVOC does not perform autopsies of forensic cases. In Brazil, an autopsy is mandatory when the nontraumatic cause of death is unclear due to lack of medical assistance or insufficient information before death and at no charge to the family. All autopsies were performed by pathologists assisted by nationally certified technicians [10].

Cases were randomly selected on weekdays between 7 a.m. and 5 p.m. Family members were asked for consent to participate in the study while waiting for the autopsy to complete. After consent was obtained, clinical and functional interviews were privately conducted.

The inclusion criteria for the BAS-USP were age 50 years and older at the time of death and the next of kin being a knowledgeable informant with at least weekly contact with the deceased. We excluded individuals for whom the informant provided conflicting information during the clinical interview. The exclusion criteria for BAS-USP were as follows: (i) brain tissue unsuitable for neuropathological analyses (e.g., cerebrospinal fluid (CSF) pH<6.5 or significant acute brain lesions, such as hemorrhages or tumors); and (ii) inconsistent clinical data provided by the informant. All the BAS-USP protocols, informed consent forms, and procedures followed international and Brazilian regulations for research involving humans [5, 10, 11] and were approved by the local and federal research committees.

### Full-body autopsy reports

A pathologist identified the CoD by full-body autopsies according to established protocols [8, 12]. First, an external body inspection was performed, followed by internal examinations of the cranial cavity and thoracic and abdominal cavities. Pathologists measured the volume of fluids and blood; examined the integrity and limits of the anatomy (external appearance of the organs and their location); and detected adhesions and obliteration of the cavities, lesions, and hemorrhages according to the general principles of pathological anatomy. Samples of abnormal areas of organs such as the kidney, spleen, lung, liver, heart, and brain were collected for anatomopathological analysis. A description of how death occurred and preexisting diseases was also compiled with a close family member. The pathologists were unaware of the group assignment (individuals with MDD or no MDD) of the present study.

As death can be a multifactorial event, all autopsy reports were completed according to a hierarchical chain of events that led to a person’s death, with up to four causes related to death (CrD) and the CoD being the last event that led to the person’s death. In our analysis, we considered CoD and at least three CrD cases when present in the autopsy report were classified according to the World Health Organization International Statistical Classification of Diseases and Related Health Problems 10th Revision (ICD-10) [8, 13].

We grouped the autopsy reports for CoD and CrD into two sets of variables. One set included the diseases grouped according to the body system or condition, producing variables encompassing the categories of diseases as in the ICD-10 chapters. Examples of these diseases include neoplasms, blood cell diseases, endocrine and nutritional diseases, cardiovascular diseases, respiratory tract diseases, digestive system diseases, and genitourinary diseases. The other set comprised diseases grouped by ICD-10 subcategories. Examples of these include neoplasms of respiratory organs or neoplasms of digestive organs.

### Clinical assessment

The date and time of death, age, sex, ethnicity (white or nonwhite), and education (illiterate, 1–4 years, or 5 years or more) were collected from the full-body autopsy reports. Other information was obtained after next of kin consented, and trained gerontologists applied the semi-structured clinical and functional assessments. The clinical evaluation assessed the deceased’s lifetime history of MDD, as well as their clinical and functional status at three months before death. A validated semi-structured clinical interview evaluated demographic characteristics, neuropsychiatric symptoms, cognitive performance, and clinical medical history [14].

The diagnosis of lifetime MDD was made using the Structured Clinical Interview for DSM-IV Disorders (SCID) for Axis I, informant part [15], and confirmed according to DSM-5 criteria. Depression was diagnosed according to the presence of symptoms during the most severe episode in life. Participants were classified as having LLD when the first MDD episode occurred after 60 years of age and RD if individuals had at least two depressive episodes.

We used the informant part of the Clinical Dementia Rating scale (CDR) [16], validated for postmortem use [14], to evaluate cognitive impairment. According to previous publications, a CDR > 0.5 was considered to indicate cognitive impairment [16, 17].

### Data set

Data derived from autopsy reports and clinical assessments were integrated into a unified table format. We introduced new variables to delineate each individual’s cause of death (CoD) and causes related to death (CrD). These included seven binary numeric variables representing diseases categorized according to the chapters of the ICD-10 classification and 25 binary numeric variables comprising diseases grouped by ICD-10 subcategories, as shown in Supplementary Table 1. Each input variable denotes the presence (value = 1) or absence (value = 0) of diseases associated with the CoD or CrD of an individual according to the ICD-10 codes of the diseases in the autopsy reports.

Participants were classified based on the presence (value = 1) or absence (value = 0) of MDD according to the criteria presented in Sect. 2.3.

ML algorithms greatly benefit from balanced data for classification tasks. To achieve this balance, we employed a matching procedure to ensure an equal number of participants with MDD and controls. This approach ensured that ML algorithms had access to equally representative examples from all cases, resulting in enhanced learning outcomes. We utilized variables such as age, sex, cognitive impairment, education, and ethnicity exclusively to perform data balancing between individuals with depression and controls.

**Fig. 1:**
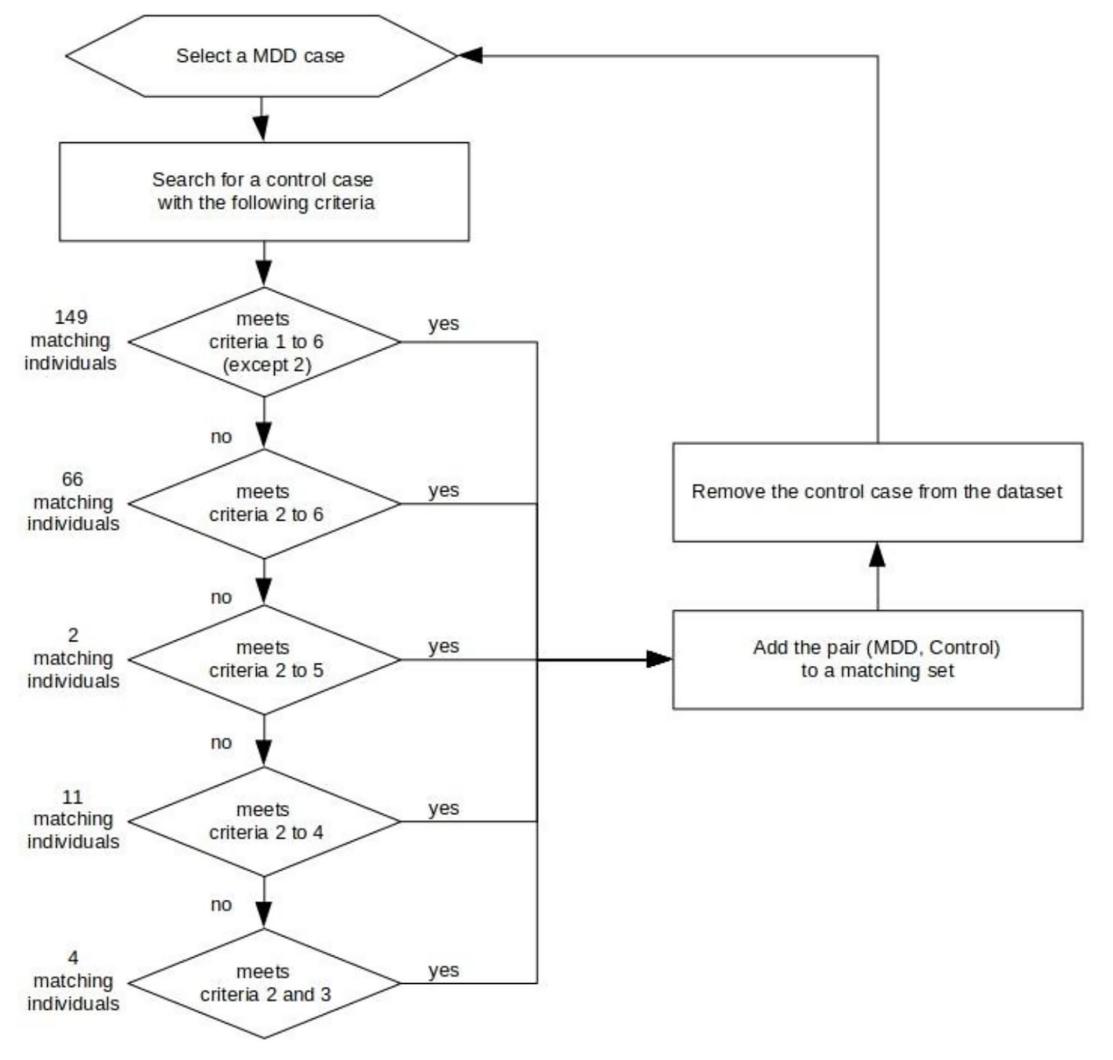
Flowchart of the algorithm used for pairing MDD individuals and controls. The matching criteria were (1) age, (2) age at death ± 4 years, (3) sex, (4) cognitive impairment, (5) education, and (6) ethnicity

Hierarchically, each MDD individual was paired with one control without MDD according to a computer-performed algorithm according to the following criteria: (1) age at death, (2) age at death ± 4 years, (3) sex, (4) cognitive impairment, (5) education, and (6) ethnicity (Fig. 1). For each participant with MDD, the algorithm initially attempted to pair them with a control case sharing the same values for age at death, sex, cognitive impairment, education, and ethnicity. If no such match was identified, the algorithm proceeded to search for a control case with age at death that could vary by plus or minus four years while maintaining all the other criteria. Following this iterative process, all 232 individuals with depression were successfully found to be matched with controls.

### Data analysis

To compare participants with depression and their paired controls, we used the Paired-Samples T-test for continuous variables and the McNemar test for categorical variables. The level of significance was set at 0.05 for two-tailed tests, followed by Bonferroni correction for multiple testing. The statistical analyses were performed using the Statistical Package for the Social Sciences (SPSS) version 20.0.

We assessed eleven established ML algorithms [18–20] to distinguish MDD, LDD and RD individuals from controls according to their CoD and CrD. The advantage of ML over classical inferential statistics is the ability to look for patterns from heterogeneous and multivariate data independent of any particular data distribution. ML methods involve making few formal assumptions, allowing the data to speak for themselves, and allowing the ability to mine structured knowledge from extensive data [21]. ML algorithms focus on prediction using general-purpose learning algorithms to discover patterns [22]. ML algorithms used ICD-10 disease category and subcategory variables as inputs to create models to distinguish participants with depression (MDD and the LLD and RD subgroups) from their controls, revealing complex multivariate and non-linear relationships between these variables. ML focus on prediction, and multivariate analysis increases sensitivity and generalizability, even with diverse data. The algorithms applied in this study included logistic regression (LR), support vector machine (SVM), K-nearest neighbor (KNN), decision tree (DT), random forest (RF), multilayer perceptron (MLP), AdaBoost (AD), gradient boosting (GB), extreme gradient boosting (XGBoost), light gradient boosting machine (LGBM), and naïve Bayes (NB) algorithms. These algorithms were implemented from the *scikit-learn* library of the Python language, version 3.7.10, with default parameters. To estimate the performance of the algorithms, we applied stratified 10-fold cross-validation, which is a method for evaluating predictive models by partitioning the dataset into a training set to create the model and a test set to assess it. In this method, data are randomly split into ten sub-samples (called folds), each with the same proportion of observations of each class (MDD, LLD, RD and their controls). This process is repeated ten times, and a different sub-sample (fold) is used as the test set. We reported the mean accuracy across all the test sets when using the different ML algorithms. Accuracy provides a straightforward way to understand how well a classification model performs and is a standard metric used to evaluate the performance of classification algorithms. Mathematically, accuracy is calculated by dividing the number of correct predictions by the total number of predictions made and multiplying by 100 to obtain a percentage. Since the number of individuals with depression (in all groups: MDD, LDD, and RD) and the respective controls are equal, accuracy is a reliable metric [23].

We conducted two separate studies using ML algorithms for MDD, LLD, and RD individuals. In the first study, we employed variables associated with disease categories as inputs for the ML algorithms. In the second study, variables specific to subcategories were utilized as inputs. As a result, each study yielded distinct outcomes for each type of depression (MDD, LLD, RD). Consequently, we generated a total of six sets of results, encompassing MDD, LDD, and RD for both category and subcategory variables.

## Results

From 2004 to 2019, we obtained data from 1,102 subjects. The mean age was 70.7 ± 11.7 years, and 500 (45.2%) individuals were female. Most participants had low educational attainment: 179 (16.2%) were illiterate, 626 (56.9%) had 1–4 years of formal education, and 294 (26.9%) had more than four years of formal education. White ethnicity was reported in 663 (60.2%) cases and 788 (71.5%) had a high-middle income. Cognitive impairment was present in 205 (18.5%) cases, and 548 (49.5%) and 362 (32.7%) had current or previous smoking and drinking habits, respectively. The ICD-10 categories and subcategory classifications for the CoD and CrD cases found in this sample can be found in Supplementary Table 1.

From this sample, 232 (21.1%) individuals had MDD and 870 (78.9%) had not. Among those in the MDD group, 190 had LLD, and 126 had RD. Each individual with MDD was paired with one control from among participants without MDD according to the algorithm; therefore, 232 controls from the group without MDD were included in further analyses. The participants with MDD and their paired controls had a mean age of 71.3 ± 11.2 years, and 268 (57.8%) were female. Educational attainment comprised 89 (19.2%) illiterate individuals, 334 (72.0%) with 1–4 years of formal education, and 35 (7.5%) with more than four years of formal education. White ethnicity was reported in 331 (67.0%) individuals, and 291 (62.7%) had a high-middle income. Cognitive impairment was present in 146 (31.5%) individuals, and 226 (48.7%) and 147 (31.7%) individuals had current or previous smoking and drinking habits, respectively. Among the comorbid antemortem clinical conditions, hypertension was found in 287 (61.9%), diabetes mellitus in 130 (28.0%), dyslipidemia in 39 (8.4%), and coronary artery disease in 108 (23.3%) individuals. A comparison of the clinical and sociodemographic variables between the MDD individuals and their controls is shown in Table 1. As expected, no differences in clinical or sociodemographic variables were found between the two groups.

**Table 1:** Comparison of clinical and sociodemographic variables among participants with major depressive disorder (MDD) and paired controls (*n* = 434)

	MDD *n* = 232	Control *n* = 232	*p*
Female, n (%)	134 (57.8)	134 (57.8)	1.000†
Age at death (years), mean (SD)	71.3 (11.3)	71.3 (11.2)	0.990^§^
Education, n (%) illiterate formal education (1–4 years) formal education (5 years or more)	45 (19.8) 165 (72.7) 17 (7.5)	44 (19.0) 169 (73.2) 18 (7.8)	0.974†
Ethnicity white, n (%)	154 (66.4)	157 (67.7)	1.000†
High-middle income, n (%)	141 (72.0)	150 (74.3)	1.000†
Cognitive impairment, n (%)	75 (32.3)	71 (30.6)	0.125†
Hypertension, n (%)	148 (63.8)	139 (60.1)	0.578†
Diabetes mellitus, n (%)	68 (29.4)	62 (26.8)	0.875†
Dyslipidemia, n (%)	17 (7.4)	22 (9.5)	0.360†
Coronary artery disease, n (%)	51 (22.5)	57 (25.0)	0.730†
Smoking, n (%)	120 (51.7)	106 (45.7)	0.775†
Drinking, n (%)	71 (30.6)	76 (32.8)	0.402†

Continuous values are presented as the means and standard deviations, and categorical values are presented as the number and percentage. †McNemar test ^§^Paired-Samples T test

MDD: major depressive disorder; SD: standard deviation

In the MDD group, examining CoD according to the ICD-10 categories, the most prevalent illnesses were diseases of the circulatory, respiratory, and digestive systems, which were present in 156 (67.2%), 31 (13.4%) and 14 (6.0%) individuals, respectively. Examining CrD according to the ICD-10 categories, the most prevalent illnesses were also diseases of the circulatory, respiratory, and digestive systems, which were present in 164 (70.7%), 31 (13.4%) and 21 (9.1%) individuals, respectively. No differences were found between the MDD individuals and their paired controls for CoD or CrD according to the ICD-10 disease categories or subcategories, as shown in Tables 2 and 3.

**Table 2:** Causes of death (CoD) for participants with major depressive disorder (MDD) and their paired controls (*n* = 464)

ICD-10 Category		MDD *n* = 232	Control *n* = 232	*p*†
Malignant neoplasms, n (%)		13 (5.6%)	15 (6.5%)	0.790
Diseases of the blood, n (%)		1 (0.4%)	3 (1.3%)	0.486
Endocrine, nutritional, and metabolic diseases, n (%)		1 (0.4%)	1 (0.4%)	1.000
Diseases of the circulatory system, n (%)		156 (67.2%)	147 (63.4%)	0.450
Diseases of the respiratory system, n (%)		31 (13.4%)	34 (14.7%)	0.790
Diseases of the digestive system, n (%)		14 (6.0%)	14 (6.0%)	1.000
Diseases of the genitourinary system, n (%)		1 (0.4%)	0	1.000
**ICD-10 Category**	**ICD-10 Subcategory**			
Malignant neoplasms	Malignant neoplasms of lip, oral cavity, pharynx, and larynx, n (%)	2 (0.9%)	0	0.530
	Malignant neoplasms of digestive organs: esophagus, stomach, small intestine, colon, rectum, n (%)	0	1 (0.4%)	1.000
	Malignant neoplasms of digestive organs: liver, biliary tract, pancreas, n (%)	2 (0.9%)	2 (0.9%)	1.000
	Malignant neoplasms of respiratory organs, n (%)	1 (0.4%)	2 (0.9%)	1.000
	Malignant neoplasm of other and ill-defined digestive organs, n (%)	2 (0.9%)	1 (0.4%)	1.000
	Malignant neoplasm of breast, cervix uteri, prostate, bladder, n (%)	0	0	-
	Malignant neoplasm without specification of site, n (%)	2 (0.9%)	2 (0.9%)	1.000
Diseases of the blood	Anemia, sickle-cell disorders, coagulation defects, n (%)	1 (0.4%)	3 (1.3%)	0.472
Endocrine, nutritional, and metabolic diseases	Diabetes mellitus, n (%)	0	0	-
	Malnutrition, n (%)	1 (0.4%)	1 (0.4%)	1.000
Diseases of the circulatory system	Hypertensive diseases, n (%)	8 (3.4%)	8 (3.4%)	1.000
	Ischemic heart diseases, n (%)	80 (34.5%)	78 (33.6%)	0.899
	Pulmonary embolism, pulmonary hypertension, n (%)	20 (8.6%)	18 (7.8%)	0.833
	Pericarditis, n (%)	11 (4.7%)	11 (4.7%)	1.000
	Cardiomyopathy, heart failure, n (%)	15 (6.5%)	15 (6.5%)	1.000
	Systemic atherosclerosis, n (%)	0	1 (0.4%)	1.000
	Aortic aneurysm, n (%)	2 (0.9%)	1 (0.4%)	1.000
Diseases of the respiratory system	Low respiratory tract infection, pneumonitis, n (%)	25 (10.8%)	31 (13.4%)	0.250
	Chronic lower respiratory diseases: chronic bronchitis, emphysema, chronic obstructive pulmonary disease, fibrosis, n (%)	3 (1.3%)	2 (0.9%)	1.000
Diseases of the digestive system	Duodenal ulcer, vascular disorder of the intestine, paralytic ileus, pancreatitis, gastrointestinal hemorrhage, n (%)	4 (1.7%)	3 (1.3%)	1.000
	Peritonitis, peritoneal adhesions, n (%)	4 (1.7%)	6 (2.6%)	0.369
	Alcoholic liver disease, toxic liver disease, hepatic failure, chronic hepatitis, cirrhosis of liver, fatty liver, n (%)	1 (0.4%)	1 (0.4%)	1.000
	Cholestatic disease, n (%)	0	0	-
Diseases of the genitourinary system	Urinary tract infection, n (%)	0	0	-
	Chronic and acute renal disease, n (%)	1 (0.4%)	0 (0.0%)	0.700

The values are given as the number of cases and percentage (%). †McNemar test

Note: ICD-10: International Statistical Classification of Diseases 10th Revision; MDD: major depressive disorder

**Table 3:** Causes related to death (CrD) for participants with major depressive disorder (MDD) and their paired controls (*n* = 464)

ICD-10 Category		MDD *n* = 232	Control *n* = 232	*p*†
Malignant neoplasms, n (%)		13 (5.6%)	15 (6.5%)	0.790
Diseases of the blood, n (%)		1 (0.4%)	3 (1.3%)	0.486
Endocrine, nutritional, and metabolic diseases, n (%)		6 (2.6%)	8 (3.4%)	0.590
Diseases of the circulatory system, n (%)		164 (70.7%)	154 (66.4%)	0.459
Diseases of the respiratory system, n (%)		31 (13.4%)	37 (15.9%)	0.530
Diseases of the digestive system, n (%)		21 (9.1%)	18 (7.8%)	0.732
Diseases of the genitourinary system, n (%)		2 (0.9%)	5 (2.2%)	0.530
**ICD-10 Category**	**ICD-10 Subcategory**			
Malignant neoplasms	Malignant neoplasms of lip, oral cavity, pharynx, and larynx, n (%)	4 (1.7%)	1 (0.4%)	0.130
	Malignant neoplasms of digestive organs: esophagus, stomach, small intestine, colon, rectum, n (%)	2 (0.9%)	7 (3.0%)	0.124
	Malignant neoplasms of digestive organs: liver, biliary tract, pancreas, n (%)	5 (2.2%)	2 (0.9%)	0.375
	Malignant neoplasms of respiratory organs, n (%)	2 (0.9%)	3 (1.3%)	1.000
	Malignant neoplasm of other and ill-defined digestive organs, n (%)	3 (1.3%)	3 (1.3%)	1.000
	Malignant neoplasm of breast, cervix uteri, prostate, bladder, n (%)	0	0	
	Malignant neoplasm without specification of site, n (%)	3 (1.3%)	2 (0.9%)	1.000
Diseases of the blood	Anemia, sickle-cell disorders, coagulation defects, n (%)	1 (0.4%)	3 (1.3%)	0.775
Endocrine, nutritional, and metabolic diseases	Diabetes mellitus, n (%)	4 (1.7%)	8 (3.4%)	0.130
	Malnutrition, n (%)	2 (0.9%)	1 (0.4%)	1.000
Diseases of the circulatory system	Hypertensive diseases, n (%)	31 (13.4%)	27 (11.6%)	0.455
	Ischemic heart diseases, n (%)	89 (38.4%)	90 (38.8%)	0.986
	Pulmonary embolism, pulmonary hypertension, n (%)	20 (8.6%)	20 (8.6%)	1.000
	Pericarditis, n (%)	12 (5.2%)	11 (4.7%)	0.933
	Cardiomyopathy, heart failure, n (%)	17 (7.3%)	15 (6.5%)	0.850
	Systemic atherosclerosis, n (%)	56 (24.1%)	54 (23.3%)	0.820
	Aortic aneurysm, n (%)	13 (5.6%)	9 (3.9%)	0.402
Diseases of the respiratory system	Low respiratory tract infection, pneumonitis, n (%)	26 (11.2%)	33 (14.2%)	0.430
	Chronic lower respiratory diseases: chronic bronchitis, emphysema, chronic obstructive pulmonary disease, fibrosis, n (%)	5 (2.2%)	6 (2.6%)	0.755
Diseases of the digestive system	Duodenal ulcer, vascular disorder of the intestine, paralytic ileus, pancreatitis, gastrointestinal hemorrhage, n (%)	6 (2.6%)	8 (3.4%)	0.450
	Peritonitis, peritoneal adhesions, n (%)	6 (2.6%)	8 (3.4%)	0.467
	Alcoholic liver disease, toxic liver disease, hepatic failure, chronic hepatitis, cirrhosis of liver, fatty liver, n (%)	3 (1.3%)	3 (1.3%)	1.000
	Cholestatic disease, n (%)	0	0	-
Diseases of the genitourinary system	Urinary tract infection, n (%)	1 (0.4%)	2 (0.9%)	0.345
	Chronic and acute renal disease, n (%)	1 (0.4%)	2 (0.9%)	0.345

The values are given as the number of cases and percentage (%). †McNemar test

Note: ICD-10: International Statistical Classification of Diseases 10th Revision; MDD: major depressive disorder

No differences were found between the LLD or RD individuals and their paired controls for CoD or CrD according to the ICD-10 disease categories, as shown in supplementary Tables 2–5. The sample size did not allow for group analysis of ICD-10 disease subcategories.

Additionally, to search for patterns that could distinguish MDD individuals from their paired controls regarding CoD and CrD, ML algorithms were used (Table 4). We explored the MDD group versus their controls and the MDD subgroups LLD and RD and their respective controls. For CoD, the performance of the ML algorithms did not allow for the distinction of MDD, LLD, or RD individuals from their controls. Average accuracies of 50.6% for MDD, 51.5% for LLD, and 48.4% for RD were found when ICD-10 disease categories were used as input variables. When considering disease subcategory as an input variable, the average accuracies were 46.5% for MDD individuals, 42.7% for LLD individuals, and 52.8% for RD individuals. For CrD, the performance of the ML algorithms also did not allow for the distinction of MDD, LLD, or RD individuals from their controls. The average accuracies were 50.5% for MDD, 47.5% for LLD, and 49.1% for RD when ICD-10 disease categories were used as input variables. When considering ICD-10 disease subcategory variables, the average accuracies were 47.8% for MDD individuals, 49.1% for LLD individuals, and 49.5% for RD individuals.

**Table 4:** Accuracy of different machine learning (ML) algorithms for differentiating causes of death (CoD) and causes related to death (CrD) among individuals with major depressive disorder (MDD) (*n* = 232), late-life depression (LLD) (*n* = 190), and recurrent depression (RD) (*n* = 126) from paired controls

	CoD using ICD-10 Categories			CoD using ICD-10 Subcategories		
ML algorithm	MDD	LLD	RD	MDD	LLD	RD
LR	51% ± 4%	51% ± 10%	49% ± 8%	46% ± 5%	41% ± 11%	49% ± 11%
SVM	51% ± 4%	51% ± 9%	47% ± 6%	45% ± 5%	41% ± 11%	59% ± 15%
KNN	52% ± 4%	49% ± 7%	50% ± 12%	51% ± 5%	50% ± 9%	56% ± 17%
DT	50% ± 4%	52% ± 10%	47% ± 6%	45% ± 5%	42% ± 10%	52% ± 15%
RF	50% ± 3%	51% ± 4%	47% ± 10%	46% ± 7%	40% ± 9%	53% ± 14%
MLP	52% ± 3%	52% ± 10%	50% ± 12%	45% ± 5%	41% ± 10%	56% ± 8%
AD	51% ± 3%	52% ± 10%	50% ± 12%	46% ± 5%	42% ± 10%	52% ± 15%
GB	51% ± 4%	52% ± 10%	49% ± 4%	46% ± 5%	42% ± 10%	52% ± 15%
XGBoost	51% ± 4%	50% ± 9%	43% ± 7%	47% ± 5%	39% ± 10%	50% ± 14%
LGBM	47% ± 3%	53% ± 10%	47% ± 6%	46% ± 5%	42% ± 9%	59% ± 20%
NB	51% ± 4%	53% ± 9%	53% ± 5%	49% ± 4%	50% ± 5%	43% ± 9%

	**CrD using ICD-10 Categories**			**CrD using ICD-10 Subcategories**		
**ML algorithm**	**MDD**	**LLD**	**RD**	**MDD**	**LLD**	**RD**
LR	51% ± 3%	44% ± 8%	48% ± 17%	48% ± 3%	48% ± 6%	46% ± 16%
SVM	50% ± 4%)	48% ± 9%	44% ± 18%	46% ± 6%	47% ± 5%	44% ± 9%
KNN	52% ± 5%	47% ± 8%	43% ± 17%	52% ± 8%	46% ± 9%	49% ± 13%
DT	51% ± 4%	47% ± 9%	48% ± 14%	47% ± 6%	50% ± 4%	47% ± 8%
RF	50% ± 4%	46% ± 11%	42% ± 16%	49% ± 6%	50% ± 8%	51% ± 9%
MLP	50% ± 3%	48% ± 9%	51% ± 13%	49% ± 7%	50% ± 6%	50% ± 8%
AD	50% ± 3%	47% ± 8%	60% ± 12%	48% ± 3%	49% ± 4%	56% ± 11%
GB	50% ± 3%	47% ± 9%	56% ± 12%	44% ± 5%	51% ± 3%	56% ± 8%
XGBoost	51% ± 4%	50% ± 10%	40% ± 16%	47% ± 3%	52% ± 6%	44% ± 14%
LGBM	50% ± 3%	49% ± 10%	54% ± 16%	46% ± 2%	47% ± 7%	58% ± 17%
NB	51% ± 3%	50% ± 8%	54% ± 12%	50% ± 3%	50% ± 3%	43% ± 13%

The values are given as the mean ± standard deviation

Note: MDD: major depressive disorder; LLD: late-life depression; RD: recurrent depression; ICD-10: Classification of Diseases and Related Health Problems 10th Revision; CoD: cause of death; ML: machine learning; LR: logistic regression; SVM: support vector machine; KNN: K-nearest neighbors; DT: decision tree; RF: random forest; MLP: multilayer perceptron; AD: AdaBoost; GB: gradient boosting; XGBoost: extreme gradient boosting; LGBM: light gradient boosting machine; NB: naïve Bayes

## Discussion

In this community-based multiethnic full-body autopsy study, 232 individuals with MDD were compared with 232 matched controls without depression. Among those in the MDD group, 190 had LLD, and 126 had RD. The most prevalent CoD and CrD were diseases of the circulatory system (69.8% and 73.3%, respectively) and diseases of the respiratory system (15.0% and 15.7%, respectively). Using McNemar’s test for paired nominal data, no differences were found between controls and MDD or its subgroups LDD and RD. We also applied several well-established ML algorithms [18–20, 23–25] to look for patterns that could distinguish individuals with and without MDD regarding CoD and CrD. Similar analyses were also carried out for LLD and RD. Despite extensive exploration of the ML algorithms, they were unable to accurately distinguish between MDD cases and controls based on CoD, indicating that CoD does not predict whether an individual belonged to the MDD or the control group.

ML algorithms are usually powerful instruments for multifactorial analysis [26] because they have the ability to mine structured knowledge from extensive data [21]. In our study, the generated ML models reached an overall average accuracy of 48.9%. This finding indicates that the performance of the models was comparable to that of random chance. Standard statistical analysis was also performed using the McNemar’s test; again, no differences were found. To further explore the current negative results, the present study involved a broad investigation of CoD, analyzing not only the direct CoD but also at least three additional CrD when reported at autopsy to increase the likelihood of finding associations with morbid conditions [13]. In our extensive exploration, we considered the autopsy reports of both the disease groups in the ICD-10 chapters to increase the power and the specific ICD-10 groups where the number of occurrences was smaller. Finally, we analyzed MDD individuals and individuals in other groups within the MDD, LLD and RD groups, and no associations were found. Discussing negative results such as ours brings us challenges, as we often face publication bias favoring positive associations [2].

No published studies analyzed CoD according to autopsy information in subjects with depression that we could compare with our negative results. Autopsy reports can provide precise information on disease pathology and account for underreported comorbid conditions. The disadvantages are that these procedures are time-consuming and are usually performed only for individuals who died. Published studies on CoD are based on health databases and death reports and not necessarily performed using full-body autopsy procedures. An extensive review of CoD showed that people with MDD were at increased risk for cardiovascular diseases [27]. However, according to an umbrella review, the evidence for causal associations between depression and all-cause mortality is still inconclusive [1]. This review systematically collected and assessed systematic reviews and meta-analyses. The credibility of each association was graded with standard approaches in the following categories: convincing, highly suggestive, suggestive, weak evidence, and nonsignificant associations. In this umbrella review, no associations between depression and CoD met the criteria for convincing evidence, while only four associations, namely, between depression and mortality in cancer individuals, heart failure, and acute myocardial infarction, were strongly suggestive of an association with depression. Nevertheless, their sensitivity analyses indicated that differences in case ascertainment of depression and the lack of proper adjustment for confounding variables and other major risk factors could render several associations supported by lower levels of evidence. Therefore, this review suggested that causal inferences between depression and CoD across distinct populations did not appear to be as conclusive as once thought, and this finding supports our negative results. Adjusting for age and sex, for instance, was considered essential. When only studies that controlled for age and sex were considered, the association between depression and CoD in cancer was no longer supported by highly suggestive evidence. Furthermore, no association was highly suggestive when only studies that employed structured or semi-structured diagnostic interviews were analyzed, which was the case in our study.

Like our study, a population-based matched cohort study covering 99% of Taiwan’s population [28] revealed no association between depression and microvascular complications, mortality due to cardiovascular diseases or diabetes mellitus. In a systematic review and meta-analysis of comorbid depression in people with diabetes, a significant association of cardiac events was found between people with depression and type 2 diabetes compared to those with type 2 diabetes alone [29]. Nonetheless, the heterogeneity was high, particularly for type 1 diabetes. The associations between cardiovascular death and depression also vary [27]. In a community sample of individuals aged 55 to 85 years, similar to our study, there was no evidence of a more significant adverse cardiac effect of depression in individuals with heart disease [30]. Interestingly, the risk of cardiac mortality was greater in more severe cases than in mild cases, as found in another study of late-onset depression [31]. This may partially explain our negative results because, in our study, most depression cases were community-based and did not come from specialized settings. Very few studies have specifically compared early-onset depression with late-onset depression for the prediction of mortality, which could help improve the prevention and treatment of different subtypes of depression and the prevention of all-cause and cardiovascular mortality [3]. Regarding prospective cohort studies, a meta-analysis suggested that late-life depression can be associated with 34 and 31% higher risk of all-cause mortality and cardiovascular mortality, respectively; however, the observed associations were subject to considerable heterogeneity across studies [3]. One limitation of the available literature is the relative scarcity of studies that use physician diagnoses to assess depression. Moreover, even among the studies that defined depression based on standardized scales, the cutoff points varied [3]. Finally, one of the few studies investigating the effects of depression treatment in older adults in primary care has shown a decrease in mortality risk [32]. For other conditions, such as cancer and diabetes mellitus, it remains unclear whether prevention and treatment of depression may increase overall survival [1]. Furthermore, interventions aimed at promoting a healthy lifestyle as well as proper care of comorbidities in those with depression may also lead to a decrease in all-cause mortality [1, 4, 33]. However, the impact of these interventions at the individual, societal, and health system levels on all-cause survival warrants further investigation [1]. The differences among studies can also be attributed to other methodological aspects, such as sample size and characteristics, number of deaths and follow-up periods, adjustment for mental disorders, and health behaviors [2].

The strengths of our study include the large sample size of community-based older adults and the diverse population in terms of ethnicity, educational background, and average income. We used standardized scales to evaluate the presence of depression. As a limitation of our study, the use of retrospectively collected informant-reported data is a concern, as informants can be unaware of some treatments and disorders of the deceased and may generate a bias towards more severe cases, particularly for MDD. For this community sample study, we might have selected more severe MDD cases where the functional impairment was severe enough to be noted by the informant or required medical support. More severe MDD cases are probably associated with more significant medical burden and, therefore, could have generated bias toward positive association to some causes of death, which was not the case in our study. To overcome the limitation of retrospectively collected informant-reported data, the results of clinical interviews with informants used in this study were validated in clinical settings [14]. To increase the reliability of these data, we included only participants who had at least weekly contact with the informant and excluded individuals when the informant provided conflicting information during the clinical interview. Gathering data from deceased individuals who underwent autopsy introduces bias and restricts the generalizability of findings. For example, slowly progressive incurable affections like some types of cancer diagnosed when an individual is still alive might lead to death, and no autopsy might be required in such cases once the cause of death is known. Therefore, some morbid conditions might be excluded in an autopsy study. However, comparing our findings with data of death causes from the general population in Brazil [34], we observed similar relative prevalence for circulatory system diseases (which were the most prevalent), followed by respiratory infections, neoplasms, and digestive system diseases. Similarly, the lifetime MDD prevalence for older adults was in accordance with the Brazilian population [35]. Socio-economic factors may also introduce bias. For example, the moment of the interview may be particularly challenging for informants. Only those feeling comfortable performing the interview provided Informed Consent, and, per ethics protocol, they could leave the study at any moment, including in the middle of the interview. As a result, we may have introduced bias in data collection through the informants because they might not be representative of the entire population of deceased individuals who need an autopsy. Therefore, cases of unexpected or sudden death (e.g., cardiovascular) where the family members experience a more severe mourning process might be less prevalent in our sample. Despite autopsies being conducted at no cost to the family in Brazil, our sample may have been skewed towards individuals of lower socioeconomic status. This could be attributed to the fact that high-income individuals (approximately 3% of the population) [36] typically have better access to medical care prior to death and may not require autopsies. However, it is unlikely that this led to a bias towards fewer cases of depression, as depression affects individuals across all income brackets. Furthermore, the absence of traumatic causes of death in our sample, including suicide, represents another source of bias. Approximately 2–3% of individuals with MDD die by suicide [37, 38], yet these severe cases could not be included in our study because they are directed to another institute. Another limitation of our study was the sample size for diseases other than cardiovascular or respiratory diseases and the RD subgroup. To draw firmer conclusions regarding causal associations between depression and CoD, further prospective and collaborative studies with transparent priori-defined protocols and proper multivariable adjustment to confounders and other essential risk determinants for mortality are warranted [1]. Finally, our study analyzed CoD in older adults in accordance with the life expectancy of this population, which is 72.3 years [36], however this might not be representative of other age ranges.

## Conclusions

Our analysis revealed that autopsy-confirmed CoD and CrD exhibited remarkable similarities between individuals with different forms of depression (MDD, LLD, and RD) and their matched controls, underscoring the existing heterogeneity in the literature. These results corroborate previous studies in the literature that did not find specific causes of death in people with MDD. Future research should prioritize prospective studies with varying age ranges, encompassing more severe manifestations of depression, and larger sample sizes, particularly in the context of CoD related to cancer.

### Electronic supplementary material

Below is the link to the electronic supplementary material.

Supplementary Material 1

## Data Availability

The datasets used and/or analyzed during the current study are available from the corresponding author upon reasonable request.
