# Supplementary material for: Causes of death in individuals with lifetime major depression: a comprehensive machine learning analysis from a community-based autopsy center
Source: BMC Psychiatry. 2024 Jul 24;24:531. doi: 10.1186/s12888-024-05946-2 (PMC11271064; doi:10.1186/s12888-024-05946-2)
Supplement: Supplementary file 1 — Supplementary Material 1 [file 12888_2024_5946_MOESM1_ESM.docx]

**Supplementary Table 1:** Categories and subcategories from the Classification of Diseases and Related Health Problems 10^th^ Revision (ICD-10) that were used to classify the cause of death or the causes related to death (n=1,102).

| **ICD-10 Category** | **ICD-10 Subcategory** | **ICD-10 coding** |
| --- | --- | --- |
| Malignant neoplasms | Malignant neoplasms of lip, oral cavity, pharynx, and larynx | C00, C01, C02, C10, C12, C14, C32 |
|  | Malignant neoplasms of digestive organs: esophagus, stomach, small intestine, colon, rectum | C15, C16, C17, C18, C20 |
|  | Malignant neoplasms of digestive organs: liver, biliary tract, pancreas | C22, C24, C25 |
|  | Malignant neoplasms of respiratory organs | C33, C34 |
|  | Malignant neoplasm of other and ill-defined digestive organs | C26, C38, C41, C56, C64, C76, C77, C78, C79 |
|  | Malignant neoplasm of breast, cervix uteri, prostate, bladder | C50, C53, C61, C67 |
|  | Malignant neoplasm without specification of site | C80 |
| Diseases of the blood | Anemia, sickle-cell disorders, coagulation defects | D50, D53, D57, D64, D68 |
| Endocrine, nutritional, and metabolic diseases | Diabetes mellitus | E14 |
|  | Malnutrition | E43, E44, E46 |
| Diseases of the circulatory system | Hypertensive diseases | I10, I11, I12 |
|  | Ischemic heart diseases | I20, I21, I22, I23, I24, I25 |
|  | Pulmonary embolism, pulmonary hypertension | I26, I27 |
|  | Pericarditis | I30, I31 |
|  | Cardiomyopathy, heart failure | I42, I50 |
|  | Systemic atherosclerosis | I70 |
|  | Aortic aneurysm | I71 |
| Diseases of the respiratory system | Low respiratory tract infection, pneumonitis | J06, J18, J20, J21, J34, J41, J69, J85, J86 |
|  | Chronic lower respiratory diseases: chronic bronchitis, emphysema, chronic obstructive pulmonary disease, fibrosis | J42, J43, J44, J84, J96 |
| Diseases of the digestive system | Duodenal ulcer, vascular disorder of the intestine, paralytic ileus, pancreatitis, gastrointestinal hemorrhage | K26, K55, K56, K85, K86, K92 |
|  | Peritonitis, peritoneal adhesions | K65, K66 |
|  | Alcoholic liver disease, toxic liver disease, hepatic failure, chronic hepatitis, cirrhosis of liver, fatty liver | K70, K71, K72, K73, K74, K76 |
|  | Cholestatic disease | K80, K81, K83 |
| Diseases of the genitourinary system | Urinary tract infection | N10, N11, N30, N39 |
|  | Chronic and acute renal disease | N03, N08, N12, N15, N17, N18, N25, N35 |

ICD-10: Classification of Diseases and Related Health Problems 10^th^ Revision

**Supplementary Table 2** Causes of death (CoD) for participants with late-life depression (LLD) and their paired controls (n=380)

| **ICD-10 Category** | LDD  n=190 | Control  n=190 | p† |
| --- | --- | --- | --- |
| Malignant neoplasms, n (%) | 7 (3.7%) | 8 (4.2%) | 0.824 |
| Diseases of the blood, n (%) | 1 (0.4%) | 3 (1.3%) | 0.486 |
| Endocrine, nutritional, and metabolic diseases, n (%) | 1 (0.5%) | 1 (0.5%) | 1.000 |
| Diseases of the circulatory system, n (%) | 127 (67.6%) | 122 (63.5%) | 0.411 |
| Diseases of the respiratory system, n (%) | 29 (15.4%) | 29 (15.4%) | 1.000 |
| Diseases of the digestive system, n (%) | 12 (6.3%) | 12 (6.3%) | 1.000 |
| Diseases of the genitourinary system, n (%) | 1 (0.5%) | 0 | 1.000 |

The values are given as the number of cases and percentage (%). †McNemar test.

Note: ICD-10: International Statistical Classification of Diseases 10^th^ Revision; LLD: late-life depression.

**Supplementary Table 3** Causes related to death (CrD) for participants with late-life depression (LLD) and their paired controls (n=380)

| **ICD-10 Category** | LDD  n=190 | Control  n=190 | p† |
| --- | --- | --- | --- |
| Malignant neoplasms, n (%) | 8 (4.3%) | 15 (7.8%) | 0.146 |
| Diseases of the blood, n (%) | 1 (0.5%) | 2 (1.0%) | 1.000 |
| Endocrine, nutritional, and metabolic diseases, n (%) | 1 (0.5%) | 2 (1.0%) | 1.000 |
| Diseases of the circulatory system, n (%) | 135 (71.8%) | 128 (66.7%) | 0.278 |
| Diseases of the respiratory system, n (%) | 29 (15.4%) | 31 (16.1%) | 0.847 |
| Diseases of the digestive system, n (%) | 16 (8.5%) | 15 (7.8%) | 1.000 |
| Diseases of the genitourinary system, n (%) | 3 (1.6%) | 2 (1.1%) | 1.000 |

The values are given as the number of cases and percentage (%). †McNemar test.

Note: ICD-10: International Statistical Classification of Diseases 10^th^ Revision; LLD: late-life depression.

**Supplementary Table 4** Causes of death (CoD) for participants with recurrent depression (RD) and their paired controls (n=252)

| **ICD-10 Category** | RD  n=126 | Control  n=126 | p† |
| --- | --- | --- | --- |
| Malignant neoplasms, n (%) | 6 (4.8%) | 5 (3.9%) | 0.737 |
| Diseases of the blood, n (%) | 0 | 1 (0.8%) | 1.000 |
| Endocrine, nutritional, and metabolic diseases, n (%) | 0 | 1 (0.8%) | 1.000 |
| Diseases of the circulatory system, n (%) | 85 (68.0%) | 74 (58.3%) | 0.109 |
| Diseases of the respiratory system, n (%) | 14 (11.2%) | 22 (17.3%) | 0.165 |
| Diseases of the digestive system, n (%) | 8 (6.4%) | 11 (8.7%) | 0.497 |
| Diseases of the genitourinary system, n (%) | 0 | 0 | - |

The values are given as the number of cases and percentage (%). †McNemar test.

Note: ICD-10: International Statistical Classification of Diseases 10^th^ Revision; RD: recurrent depression.

**Supplementary Table 5** Causes related to death (CrD) for participants with recurrent depression (RD) and their paired controls (n=252)

| **ICD-10 Category** | RD  n=126 | Control  n=126 | p† |
| --- | --- | --- | --- |
| Malignant neoplasms, n (%) | 8 (6.4%) | 9 (7.1%) | 0.828 |
| Diseases of the blood, n (%) | 0 | 1 (0.8%) | 1.000 |
| Endocrine, nutritional, and metabolic diseases, n (%) | 2 (1.6%) | 4 (3.1%) | 0.684 |
| Diseases of the circulatory system, n (%) | 88 (70.4%) | 79 (62.2%) | 0.169 |
| Diseases of the respiratory system, n (%) | 14 (11.2%) | 23 (18.1%) | 0.121 |
| Diseases of the digestive system, n (%) | 11 (8.8%) | 13 (10.2%) | 0.698 |
| Diseases of the genitourinary system, n (%) | 1 (0.8%) | 2 (1.6%) | 1.000 |

The values are given as the number of cases and percentage (%). †McNemar test.

Note: ICD-10: International Statistical Classification of Diseases 10^th^ Revision; RD: recurrent depression.
